# Algae as Reservoirs for Coral Pathogens

**DOI:** 10.1371/journal.pone.0069717

**Published:** 2013-07-31

**Authors:** Michael J. Sweet, John C. Bythell, Maggy M. Nugues

**Affiliations:** 1 School of Biology, Ridley Building, Newcastle University, Newcastle upon Tyne, United Kingdom; 2 Laboratoire d'Excellence ‘CORAIL’ and USR 3278 CRIOBE EPHE-CNRS, CBETM de l'Université de Perpignan, Perpignan, France; University of New South Wales, Australia

## Abstract

Benthic algae are associated with coral death in the form of stress and disease. It's been proposed that they release exudates, which facilitate invasion of potentially pathogenic microbes at the coral-algal interface, resulting in coral disease. However, the original source of these pathogens remains unknown. This study examined the ability of benthic algae to act as reservoirs of coral pathogens by characterizing surface associated microbes associated with major Caribbean and Indo-Pacific algal species/types and by comparing them to potential pathogens of two dominant coral diseases: White Syndrome (WS) in the Indo-Pacific and Yellow Band Disease (YBD) in the Caribbean. Coral and algal sampling was conducted simultaneously at the same sites to avoid spatial effects. Potential pathogens were defined as those absent or rare in healthy corals, increasing in abundance in healthy tissues adjacent to a disease lesion, and dominant in disease lesions. Potentially pathogenic bacteria were detected in both WS and YBD and were also present within the majority of algal species/types (54 and 100% for WS and YBD respectively). Pathogenic ciliates were associated only with WS and not YBD lesions and these were also present in 36% of the Indo-Pacific algal species. Although potential pathogens were associated with many algal species, their presence was inconsistent among replicate algal samples and detection rates were relatively low, suggestive of low density and occurrence. At the community level, coral-associated microbes irrespective of the health of their host differed from algal-associated microbes, supporting that algae and corals have distinctive microbial communities associated with their tissue. We conclude that benthic algae are common reservoirs for a variety of different potential coral pathogens. However, algal-associated microbes alone are unlikely to cause coral death. Initial damage or stress to the coral via other competitive mechanisms is most likely a prerequisite to potential transmission of these pathogens.

## Introduction

Coral diseases have contributed to the regional collapse of important reef-building species worldwide [1]–[3]. Despite a concerted global initiative to characterise coral diseases over the past two decades, the causes of disease outbreaks remain largely unknown [4]. Their impacts are continuing to increase in number and spatial extent, raising hypotheses that they could be associated with large-scale environmental stressors such as rising ocean temperature or acidification [5], [6]. Additionally, nutrient pollution has been shown to increase the spread of several coral diseases and thus could exacerbate disease outbreaks locally [7], [8]. A recent but still largely unexplored hypothesis is that coral diseases may be enhanced by the increased abundance of benthic algae which reefs are experiencing on regional and global scales [9]–[12].

Competition between corals and benthic algae is frequent on reefs especially under conditions of reduced grazing and increased nutrients, a change which favors the growth of macroalgae and turf algae [13], [14]. The incidence of coral disease has been found to be positively correlated with increasing algal cover [15]–[18], and the experimental link between direct algal contact and coral disease has been established in the field [19], [20]. However, there is no clear mechanistic link between algae and coral diseases. Contact between corals and algae commonly cause a suite of stresses for corals, such as localized tissue death, reductions in tissue thickness and zooxanthellae density and lower photosynthetic efficiency often resulting in bleaching [21], [22], all of which could increase coral susceptibility to disease pathogens. Current evidence suggests that algae have the ability to directly harm corals in three principal ways; 1) shading, 2) abrasion resulting in a physical injury, and 3) the release of poisonous allelochemicals (reviewed by [23]). There are also numerous indirect ways the algae have been shown to have an effect, notably: 4) via the release of primary metabolites, which has been shown to stimulate microbial activity at the coral/algal interface [24]–[26], 5) via the release of secondary metabolites which alter the coral associated microbial community [27]–[30], 6) by attracting corallivores, which cause increased coral mortality [31], and lastly 7) by acting as a vector for coral disease pathogens [19]. In contrast to the above mentioned studies which illustrate numerous direct and indirect effects of algae on coral [32], showed that the presence of macro algae adjacent to corals had no observable effect on coral health and disease prevalence at least in relation to Yellow Band Disease within the Caribbean. However, in their experiments, macroalgae were not directly touching the coral, which suggests that water borne algal exudates alone are not sufficient to illicit a stress response on the coral.

Regardless of the main trigger for coral death by algae, with regard do disease onset the source of coral pathogens remains unknown. A possible mechanism for this, is that algae may act as vectors of coral pathogens, transmitting them from their surfaces to corals via direct contact [19]. showed that direct contact of the green algae *Halimeda opuntia* to apparently healthy corals could trigger white plague disease and found the bacterial pathogen, *Aurantimonas coralicida* (the proposed causal agent of WP Type II disease, [33]) to be associated with *H. opuntia*. Furthermore, more recently [20], showed that, under experimental conditions, contact between the coral *Acropora pulchra* and the green filamentous macroalgae *Chlorodesmis fastigiata* often resulted in a ciliate infection on the coral. However, in both cases, it has not yet been demonstrated whether the pathogens were directly transmitted by the algae.

As a first step, it seems relevant to study the availability of potential coral pathogens associated with algal surfaces and to determine which of these are also associated with specific coral diseases. Many species of bacteria have been accredited to causing specific coral diseases [33]–[39], in particular members from the genus *Vibrio* such as *V. shiloi*, *V. coralliilyticus* and *V. harveyi*, along with others such as *Aurantimonas corallicida* [40]. recently characterized the bacterial communities on the surface of benthic algae and found that some matched with sequences related to bacteria associated with coral disease states highlighted by other studies. However, no study has simultaneously investigated the dominant bacterial communities associated with both algae and healthy/diseased corals from the same reefs using the same methodology. As the microbial communities of the coral holobiont vary both spatially [41] and temporally [42], it is important that samples are collected at the same time, extracted in the same manor and analysed using the same technique. In addition to potential bacterial pathogens, other microorganisms such as ciliates have been shown to be associated with many coral diseases [43]–[45]. Three species, two from the genus *Philaster* and a *Varistrombidium*, have been shown to ingest coral algal symbionts and it has been hypothesized that they cause the pathology of the diseases known collectively as White Syndrome [45]. Despite their importance in many ecosystems, ciliates have received considerably little attention and their abundance on algal surfaces has not been studied to date.

Here we examine the bacterial and ciliate communities associated with various macroalgal species and turf algae using culture independent techniques (16S and 18S rRNA genes) and compare them to potential pathogens of two diseases dominating the Indo-Pacific and Caribbean regions: White Syndrome (WS) and Yellow Band Disease (YBD) respectively. Several *Vibrios* have been proposed as WS pathogens [37], [39]. However, a recent study suggest that ciliates are largely responsible for the macroscopic signs of the disease [45]. YBD has no named causal agent to date, however the Vibrio ‘core group’, similar to species reported to cause WS in the Indo-Pacific, has been shown to play a role in the degeneration and deformation of the coral algal symbionts in the disease [35], [46]. ‘Potential pathogens’ in this study were defined as those absent or rare in healthy corals, increasing in abundance in apparently healthy tissues (those adjacent to the disease lesion), and dominant in the disease lesion itself [45]. These potential pathogens were identified by examining coral disease-associated microbes on the same reefs, at the same time and using the same procedures as those associated with various algae. We were interested in detecting only those microbes associated with the surface of the algal species sampled as these will be the microbes which will most likely come into contact with the coral interface and therefore most likely pass from algae to coral, hence we utilized a modified DGGE-based technique known to only detect the dominant members of the microbial communities allowing for a larger sample size to be studied at relatively lower cost techniques such as high throughput sequencing.

## Methods

### Sample collection

**Figure 1:**
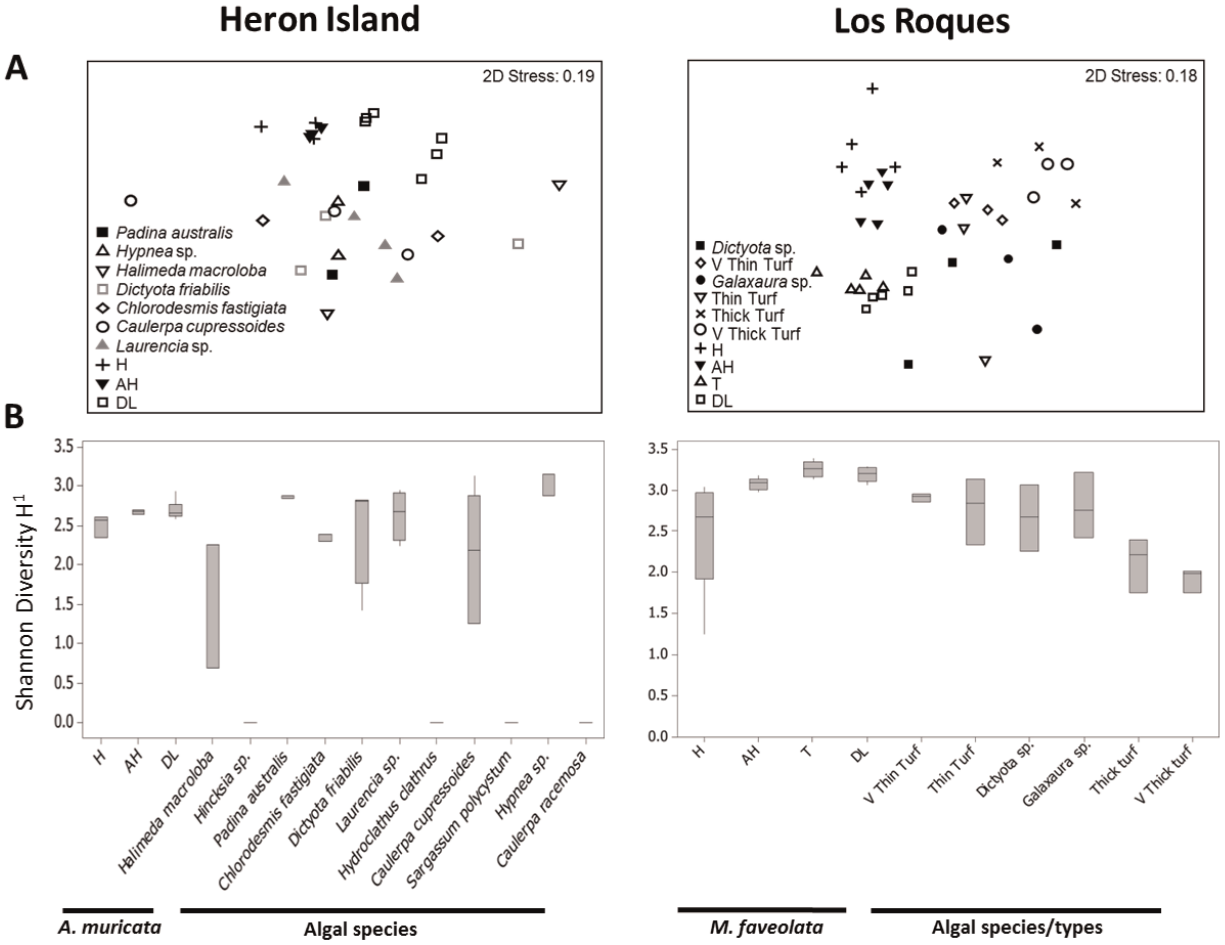
Bacterial 16S rRNA gene communities associated with various coral and algal samples at Heron Island and Los Roques. **A**) Multidimensional scaling (MDS) plot showing changes in bacterial communities. **B**) Shannon-Wiener Diversity Index. The middle bars are medians, boxes show quartiles, whiskers show 95% CI of data. H = non diseased, AH = apparently healthy, DL = disease lesions, T = transition.

Samples of corals and algae were collected at Heron Island, on the Great Barrier Reef in the Indo-Pacific and at Los Roques, Venezuela in the Caribbean in accordance with collection permits issued by the Great Barrier Reef Marine Park Authority (GBRMPA) and the Instituto Nacional de Parques (INPAQUES), respectively. Coral collection procedure follows that of [45]. At Heron Island, colonies of *Acropora muricata*, suffering from signs of White Syndrome, were tagged and monitored for 4 days to follow the progression of disease lesions. Samples were collected from actively progressing disease lesions (DL; n = 3) and apparently healthy tissue (AH; n = 3) ∼1 cm away from the DL interface. Non-diseased coral colonies (ND; n = 3) were sampled as controls. Alongside these coral samples, eleven algal species (n = 3 replicates per species, listed in Fig. 1) were collected. These were common to abundant at Heron Island, were observed to encounter corals and represented a wide range of morphological and taxonomic groups. All samples were collected *in situ* into sterile 50 ml falcon tubes and transported back to the laboratories. The water was then replaced with 100% EtOH and stored at −20°C until extraction. Coral and algal samples were centrifuged at 20,000 g to concentrate loosely associated microbes and surface mucus layer (of the corals specifically) [47]. Although this technique is a novel approach to study both coral and algal microbial associates, the presence of coral DNA in the extracted samples illustrates that this technique successfully samples both tissue and surface mucus of corals. All samples were then vacuum centrifuged to reduce the volume of EtOH/homogenized tissues to 10 ml in each sample. A subset of the homogenized tissue and mucus was then sampled and weighed to allow for standardization between sample types.

In Los Roques, *Montastraea faveolata* showing signs of YBD were tagged and monitored as above. Coral samples were taken from the same areas, with the addition of a further set of samples at the transition (bleached) zone (T; n = 3) of the disease lesion. Two algal species and four turf algal types (n = 3 replicates per type) were collected alongside the coral samples: *Dictyota* sp., *Galaxaura* sp., very thin turf (<2 mm), thin turf (2–<5 mm), thick turf (5–10 mm) and very thick turf (>1 cm). Turf algae consisted of diminutive mixed assemblages of filamentous algae, juvenile macroalgae and cyanobacteria [48]. Turf thickness was measured *in situ* with calipers and samples were scrapped using a sterile scalpel blade into 50 ml falcon tubes and transported to the surface. Samples were processed and stored similar to those taken from Heron Island. All algal replicates were taken at least 10 meters apart from each other to minimize the chance of individual variation within species.

### DNA extraction, DGGE and sequence analysis

All samples were extracted using the Qiagen DNeasy Blood and Tissue Kit, spin column protocol [49]. A portion of the bacterial 16S rRNA gene was amplified using universal eubacterial primers (GC-357F) (5′- CGCCCGCCGCGCGCGGCGGGCGGGGCGGGGGCAGCACGGGGGG-CCTACGGGAGGCAGCAG-3′) and (518R) (5′-ATTACCGCGGCTGCTGG-3′). All reactions were performed using a Hybaid PCR Express thermal cycler. PCR and DGGE were carried out as described by [49]. To identify the dominant DGGE bands across samples, representative bands (n = 50) were excised and sequenced to account for known DGGE artifacts such as heteroduplexes [50]. Excised bands were left overnight in Sigma molecular grade water, vacuum centrifuged, re-amplified with primers 357F and 518R, labeled using Big Dye (Applied biosystems) transformation sequence kit and sent to Genevision (Newcastle University, UK) for sequencing. Bacterial operational taxonomic units (OTUs) were defined from DGGE band-matching analysis using BioNumerics 3.5 (Applied Maths BVBA) as described by [49]. From the same extracted samples as above, ciliate 18S rRNA genes were amplified directly using the primers CilF (5′-TGGTAGTGTATTGGACWACCA-3′) CilDGGE-r (5′TGAAAACATCCTTGGCAACTG-3′). PCR and DGGEs were carried out as described in [45].

### Statistical analysis

DGGE images were analyzed using BioNumerics version 3.5. Quantification of samples is based on the densitometric curves extracted from the banding patterns. Minimum profiling was set at 10% which allows elevation of the band with respect to the background noise of the gel. Any bands specified as ‘uncertain’ were removed at this step. This analysis allows for both the presence/absence of the OTUs and relative intensity to be analyzed. A one-way analysis of similarity (ANOSIM) based on Bray-Curtis similarities of both band intensity patterns and presence/absence only were performed to test for differences between DGGE profiles of the bacterial 16S rRNA and ciliate 18S rRNA gene assemblages associated with different coral health states and the algal species using PRIMER v6 [51]. Pairwise comparisons within ANOSIM were used to contrast between specific sample types [52]. Non-metric multidimensional scaling (MDS) was used to visualize variations in bacterial and ciliate communities among the different samples. Shannon-Weiner diversity (H') was calculated as an estimate of diversity and analysed using PERMANOVA followed by pairwise tests.

## Results

### 16S rRNA gene bacterial community

**Figure 2:**
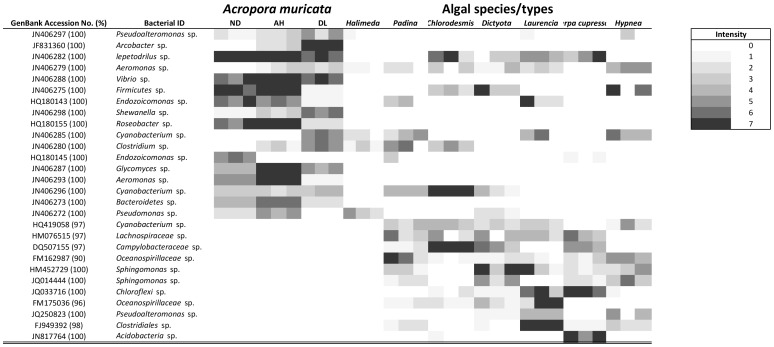
Heatmap summarizing the relative density of dominant DGGE bands according to BioNumerics software for 16S rRNA gene bacterial profiles of coral and algal samples collected in Heron Island. White blocks signifies that that ribotype was not detected in the sample. All replicates (N = 3 per sample type) have been included in this figure. ND = non diseased, AH = apparently healthy, DL = disease lesions, T = transition. For full algal species names see Fig. 1.

**Figure 3:**
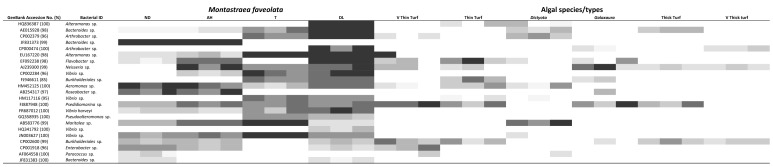
Heatmap summarizing the relative density of dominant DGGE bands according to BioNumerics for 16S rRNA gene bacterial profiles of coral (*M.faveolata*) and algal samples collected in Los Roques. All replicates (N = 3 per sample type) have been included in this table. Abbreviations as in Fig. 1 and key as in Fig. 2.

In both study sites, 16S rRNA gene bacterial communities differed among sample types (ANOSIM, Heron Island: R = 0.371, p<0.001; Los Roques: R = 0.743, p = 0.001; Figs. 1a). Coral samples regardless of host health consistently differed from algal samples regardless of species/types (Fig. 1; Tables S1 & S2). Healthy coral tissue differed more with diseased and/or transition tissue (in the case of YBD) than with apparently healthy tissue. Variation among algal samples was less (Fig. 1; Fig. 2). Bacterial communities did not differ among algal species which hosted bacteria at Heron Island (Fig. 1; Fig. 2; Table S1). At Los Roques, thick turf and very thick turf were significantly different than the other algal types (Fig. 1; Fig. 3; Table S2). Shannon-Wiener diversity varied significantly between sample types for both Heron Island and Los Roques (PERMANOVA F = 24.264, p = 0.001; F = 26.342, p = 0.001 respectively) (Tables S3 & S4). Coral-associated bacterial diversity increased with reduced health of the host (Fig. 1b). Bacterial diversity was generally lower in the algal samples when compared to the coral with the exception of *Padina australis*, *Laurencia* sp. and *Hypnea* sp. from Heron Island (Fig. 1b). However, there was no significant differences in bacterial diversity between and within both the coral and algal samples at Heron Island (Fig. 1; Table S3), and only the bacterial diversity of the transition band in corals showing signs of YBD and the lesion interface itself were significantly different to healthy coral samples at Los Roques (Fig. 1; Table S4). At Heron Island, four out of the eleven algal species (*Hincksia* sp., *Hydroclathrus clathrus*, *Sargassum polycystum* and *Caulerpa racemosa*) showed no bacterial associates, and only a few bacterial ribotypes were detected on *Halimeda macroloba* (Fig. 2). In contrast, surface-associated bacteria were present in all algal species/types at Los Roques (Fig. 3). Fewer bacteria were found on thick and very thick turf compared to thinner turf types and the two macro-algal species.

Four potential WS bacterial pathogen ribotypes (NCBI accession numbers JF831360, JN406279, JN406285 and JN406280) identified as an *Arcobacter* sp., an *Aeromonas* sp., a *Cyanobacterium* sp., and a *Clostridium* sp. respectively, were detected in coral samples at Heron Island. Three of these (*Aeromonas* sp.; *Cyanobacterium* sp. and *Clostridium* sp.) were also present in algal samples, with all three being detected on *Padina australis* and *Halimeda macroloba* (Fig. 2). Overall 6 of the 11 algal species (54%) and 18 of the 33 algal samples (54%) included potential bacterial pathogens. *Aeromonas* sp. was detected in 15 of the 33 algal samples (45%), *Cyanobacterium* in 11/33 (33%) and *Clostridium* in 8/11 (24%) (Fig. 2). A further two species, *Pseudoalteromonas* (JN406297) and *Shewanella* (JN406298) increased in abundance in the WS diseased lesion samples, but they were also present in healthy tissues. These were only detected in two algal species, *Hypnea* and *Halimeda* and only in one of the replicates of each of the algae. Out of the algae in which surface bacteria were detected for, only *Caulerpa cupressiodes* was free of these potential WS pathogens.

In Los Roques, nine bacterial ribotypes were identified as potential YBD pathogens, these included ribotypes relating to a *Burkholderiales* sp. (NCBI accession number FJ946611), a *Pseudoalteromonas* sp. (GQ358935), three *Vibrio* sp. (CP002284, HM117116 & HQ341792), a *Alteromonas* sp. (HQ836387), a *Neisseria* sp. (AJ239300) and two *Arthrobacter* sp. (CP002379 & CP000474) (Fig. 3). Six of these (the *Neisseria* sp., the *Alteromonas* sp., the two *Arthrobacter* sp., the *Burkholderiales* sp. and one of the *Vibrio* sp.) were found to be associated with algae of some kind. All algal species/types and all algal replicate samples harbored potential bacterial pathogens. *Neisseria* was the most common bacterial ribotype detected in the algal samples and was present in 5 of the 6 algal species/types (83%) and 13 of the 18 algal samples (72%). Out of the two *Arthrobacter*, one was present in *Dictyota* and very thin turf, whilst the other was present in *Galaxaura* and very thick turf.

### 18S rRNA gene ciliate assemblage

**Figure 4:**
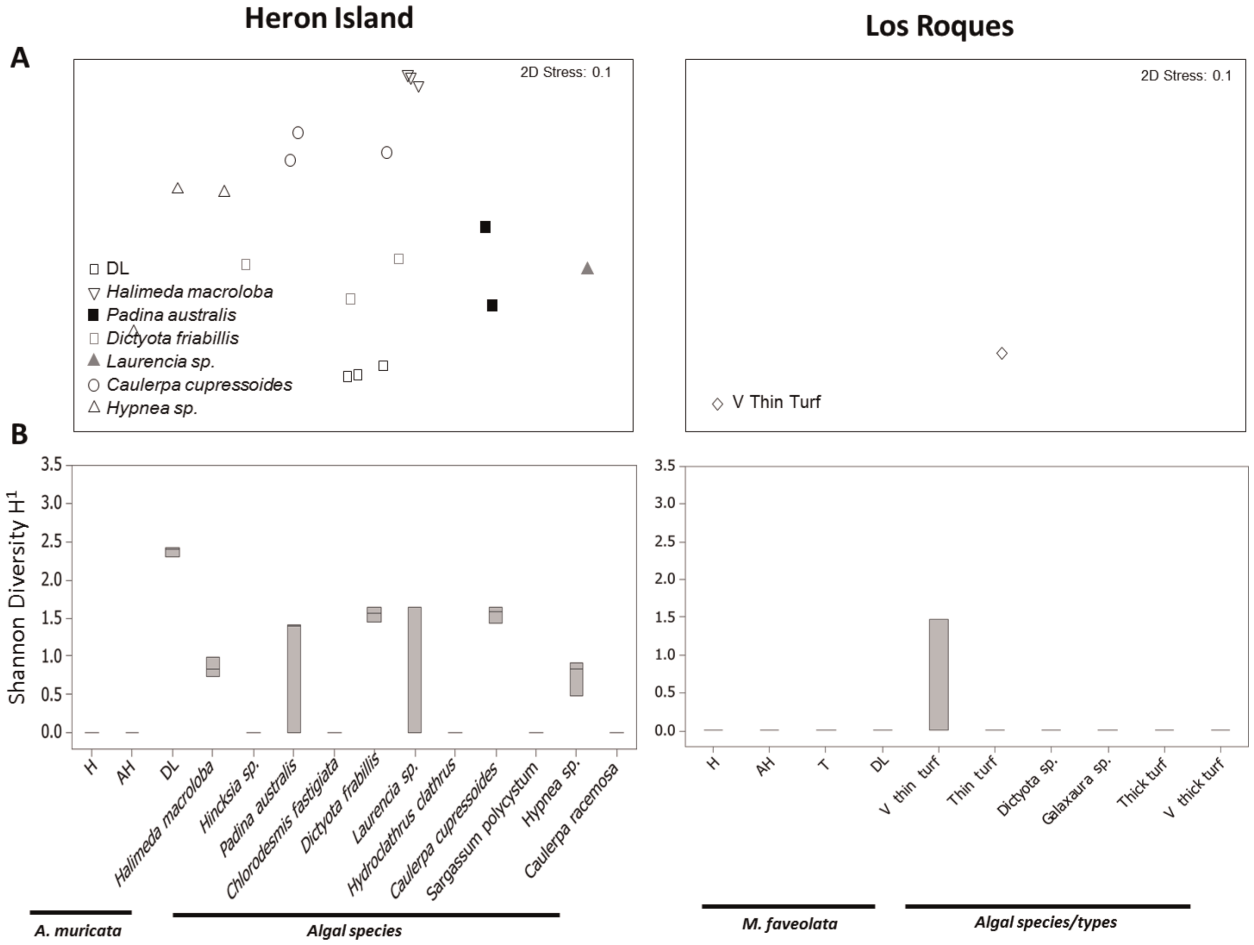
Ciliate 18S rRNA gene communities associated with various coral and algal samples at Heron Island and Los Roques. **A**) Multidimensional scaling (MDS) plot showing changes in ciliate communities. **B**) Shannon-Wiener Diversity Index. The middle bars are medians, boxes show quartiles, whiskers show 95% CI of data. Abbreviations as in Fig. 1.

**Figure 5:**
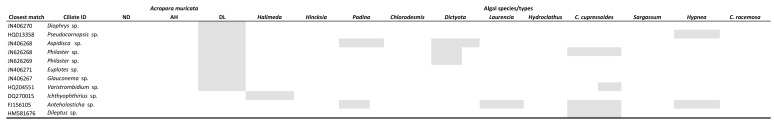
Heatmap summarizing the relative density of dominant DGGE bands according to BioNumerics for 18S rRNA gene ciliate profiles of coral (*A. muricata*) and algal samples collected in Heron Island. All replicates (N = 3 per sample type) have been included in this table. Abbreviations as in Fig. 1 and key as in Fig. 2. For full algal species names see Fig. 1.

**Figure 6:**
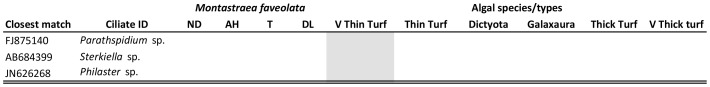
Heatmap summarizing the relative density of dominant DGGE bands according to BioNumerics for 18S rRNA gene ciliate profiles of coral (*M. faveolata*) and algal samples collected in Los Roques. All replicates (N = 3 per sample type) have been included in this table. Abbreviations as in Fig. 1 and key as in Fig. 2.

In Heron Island, ciliate assemblage associated with corals showing signs of WS differed from any other (coral or algal) sample (ANOSIM R = 0.67, p = 0.001 and pairwise tests p<0.05) (Fig. 4a; Fig. 5). These ciliates were consistently absent from healthy and apparently healthy tissues. WS associated ciliates included ribotypes similar to *Aspidisca* sp. (JN406268), *Diophrys* sp. (JN406270), *Philaster* sp. (JN626268 and JN626269), *Pseudocarnopsis* sp. (HQ013358), *Euplotes* sp. (JN406271 and HQ013357), *Glauconema* sp. (JN406267) and *Varistrombidium* sp. (HQ204551) (Fig. 5). Six out of the eleven algal species (*Halimeda macroloba*, *Padina australis*, *Dictyota friabilis*, *Laurencia* sp., *Caulerpa cupressoides*, *Hypnea* sp.) hosted ciliates (Fig. 5), with eight ciliate species being detected. Five of these ciliate species were associated with WS diseased corals: *Aspidisca* sp., the two *Philaster* sp. (JN626268 and JN626269), *Varistrombidium* sp. and *Pseudocarnopsis* sp. The other three had ribotypes related to *Anteholosticha* sp. (FJ156105), *Ichthyophthirius* sp. (DQ270015) and *Dileptus* sp. (HM581676). Four of the 11 algal species (36%) and 10 of the 33 algal samples (30%) included WS associated ciliates. In contrast to WS, ciliates were not associated with Yellow Band Disease (YBD) tissues or any other coral samples collected from Los Roques (Fig. 6), however they have been visually observed in some cases of the disease (see Video S1). Only one algae (very thin turf) showed the presence of ciliates in the Los Roques samples (Fig. 4; Fig. 6), however the diversity of ciliates associated with this algae was consistent between all replicates. Three different ciliate ribotypes were detected on very thin turf including ribotypes closely related to *Parathspidium* Sp. (FJ875140), *Sterkiella* sp. (AB684399) and a *Philaster* sp. (JN626268).

## Discussion

To determine whether benthic algae act as a source of coral pathogens, we examined dominant surface microbes (bacteria and ciliates) associated with a variety of algae species and related them, using the same non culture molecular technique (DGGE), to dominant potentially pathogenic microbes associated with two coral diseases. In the Indo Pacific, several potentially pathogenic bacteria detected in this study have previously been shown to be associated with coral disease states, such as *Vibrio* sp. (JN406288), *Arcobacter* sp. (JF831360), *Aeromonas* sp. (JN406279), *Cyanobacterium* sp. (JN406285), *Clostridium* sp. (JN406280), *Pseudoalteromonas* sp. (JN406297) and *Shewanella* (JN406298) [39], . Similarly, out of the five ciliates detected within the algal samples, three have been shown to be involved in coral diseases [45], two species from the genus *Philaster* (JN626268 and JN626269) and a *Varistrombidium* sp. (HQ204551). A further ciliate detected in the algae samples has been identified as the causal agent of an aquarium fish disease known as Ich (the ciliate *Ichthyophthirius* sp. DQ270015) [64]. In all cases except for ciliates at Los Roques which were not associated with YBD, potential bacterial and ciliate pathogens were detected in a majority of algal species or types, supporting the hypothesis that benthic algae are a common reservoir for coral disease pathogens.

A meta-analysis study reviewing coral disease associated bacteria conducted by [56], showed that, in diseased corals, the bacterial communities were consistently dominated by an increase in *Rhodobacter*, *Clostridia* and *Cyanobacteria*. In our study, the latter two orders increased in abundance in the WS samples, but were absent in YBD tissues. In contrast, although they were identified in both coral diseases representatives of *Rhodobacter* decreased in abundance in diseased tissues contrary to that reported by [56] and patterns observed for other diseases such as Black Band Disease [55], White Plague and White Band Disease [57], [58]. These contrasts between studies are likely to be brought about by the molecular profiling techniques utilized and specific primers chosen, as methodological studies have shown that primer choice can dramatically alter the end result of the bacterial assemblages [59]. This was the reasoning, at least in part, why this study analyzed the microbial diversity of both algae and corals at the same time, rather than simply relying on previous studies to highlight similarities and differences within the two community types.

Several *Vibrio* species such as *Vibrio coralliilyticus* and *V. harveyi* have been identified as potential WS pathogens by numerous previous studies [37], [39]. However, although the technique employed here (DGGE) does not bias against vibrios as demonstrated by the dominance of *Vibrio* sp. in the YBD diseased coral tissue, only one strain of vibrio closely related to *V. harveyi* (JN406288) was found in the coral samples at Heron Island. This strain was not identified as a potential pathogen since it had similar relative band intensity in both diseased and healthy tissues. This finding agrees with other studies [45], [60], which also used more stringent non culture molecular techniques such as clone libraries to show the same result. Furthermore, this ribotype was not detected on any of the algae sampled in this study.

The YBD bacterial community profiles showed similar results to that of the Indo-Pacific with numerous potential pathogens present in the coral samples. These included ribotypes related to *Neisseria* sp. (AJ239300), *Burkholderiales* sp. (FJ946611), *Pseudoalteromonas* sp. (GQ358935), *Alteromonas* sp. (HQ836387) and two *Arthrobacter* sp. (CP002379 & CP000474). All but the *Pseudoalteromonas* sp. were found to be associated with various algal species, though in relatively low dominance. Although no known causal agent has been identified for YBD to date, the disease lesion has previously been associated with a significant increase in the core *Vibrio* group at the lesion interface and in the transition band, giving rise to the disease name [35], [46]. This is consistent with results in our study whereby a large number of ribotypes closely related to different *Vibrios* were found in diseased samples. However, two of these *Vibrio* ribotypes (FR687012 & JN003627) were found in healthy samples showing that healthy tissues can harbor bacteria normally considered potentially pathogenic, a result increasingly seen in recent studies [45], [46], [58]. Two *Vibrios* (HM117116 & JN003627) were found associated with algae (*Dictyota* and thin turf) and only one of these (HM117116) was considered as a potential pathogen of YBD, being absent in healthy tissues and present in the diseased tissues.

[40] expanded the coral holobiont concept to benthic algae on the basis that, like corals, algae hosted abundant and distinctive microbial communities and that these microbes provided benefits to their host. Similarly to their study and in support of this concept, we found that dominant algal surface associated microbes were distinct from those associated with corals and varied among algal species. Interestingly certain species such as *Halimeda macroloba* exhibited low density bands as opposed to *Hypnea* sp. or *Chlorodesmis fastigiata*. Furthermore, four of the eleven algal species (*Hincksia* sp., *Hydroclathrus clathrus*, *Sargassum polycystum* and *Caulerpa Racemosa*) sampled in Heron Island showed no recoverable surface associated microbes. In our view, it remains unlikely that this result is an extraction error, as all samples were treated individually and no recoverable bacterial or ciliate sequences were detected in any of the replicate samples, however further analysis on these algae should be conducted to confirm this. In support of our results, algae commonly exhibit mucus release and tissue sloughing, they release different primary and secondary compounds and may have resident bacteria deeper within their tissues which have specific antibiotic and antifouling properties [61], [62]. Together these factors may create these distinctive microbial communities which are associated with the different algal species.

[40] also highlighted that algae have a higher diversity of bacterial OTUs than those associated with corals. In contrast, our study showed that the dominant bacterial ribotypes associated with the surface of the algae were in general no more diverse than those present on the surface of corals. In fact in most species, diversity was generally lower than the coral samples as a whole. This difference in results between the two studies may in part be methodological [40]. homogenised the algal tissue giving total bacterial diversity associated with the algal species and utilized deep sequencing techniques which allow insight into the whole bacterial community. In our study, samples of both coral and algae were centrifuged to optimize the detection of surface associated microbes and the non-culture molecular technique, DGGE, was used to characterize the dominant members of the microbial communities associated with the samples, as these will be the microbes which will most likely come into contact with the coral interface and therefore most likely pass from algae to coral. While this vastly reduces the cost of analyses, it has the disadvantage of underestimating total bacterial diversity.

Similar to that observed for bacterial associates, ciliated protozoans were detected within algae as well as diseased lesions, although only in the case of WS [45]. recently showed that four species ingest algal symbionts from corals (two from the genus *Philaster*, one *Euplotes* sp. and one *Varistrombidium* sp.). It was argued that these ciliates, particularly the two *Philaster* sp., were largely responsible for the macroscopic signs of the disease (WS), namely the sharp demarcation between apparently healthy tissues and the denuded skeleton giving rise to the name white syndrome. In this study, the two main potential agents, identified as Morph 1 and Morph 2 in [45], were found in two algal species (*D. friabilis* and *C. cupressoides*) in Heron Island. Although these ciliates were not consistently prevalent in the algae (1–2 out of the three replicates of each algal species), our study suggests that algae can act as a reservoir for ciliates pathogenic to corals and in particular involved in WS.

A recent experimental study by [20] showed that corals which were in direct contact with the algae *Chlorodesmis fastigiata* often resulted in a ciliate infection similar to Brown Band Disease. They visually investigated tufts of *C. fastigiata* at the time to see if this algae was a carrier for the ciliate pathogens, but failed to find any signs. In support, using a more rigorous molecular profiling technique, we also found no ciliates associated with the algae. These results suggest that these ciliate infections may be the result of the interaction between the algae and the coral. Such interaction commonly results in damaged coral tissue, which may attract the ciliates, in turn leading to infection. Thus, the algae may not be the source of this specific pathogen. In contrast to that of Heron, at Los Roques, only one algal type (very thin turf) harboured ciliates. Interestingly, one of the three species detected in this type was 98% similar to the same *Philaster* sp. (JN626268) associated with WS in the Indo-Pacific. Although we only studied pathogens associated with YBD in which ciliates were apparently not pathogenic, it remains to be investigated whether other Caribbean diseases, such as White Plague and White Band Disease, which share similar gross macroscopic signs with WS, could be associated with this species of ciliate.

In conclusion, this study shows that algae do contain certain potential coral pathogens. As reservoirs of these pathogens, algae therefore have the potential to act as vectors of coral diseases, transmitting pathogens to corals by direct contact. However, it has been argued that the increase in coral diseases combined with a decrease in host densities suggests opportunistic infections of compromised hosts rather than the involvement of specific pathogens is more likely [63]. It is highly probable that other factors mediated by algae such as the release of primary and secondary metabolites which cause abnormal stress or trauma to corals [24], [25], [27]–[29], are required to weaken the corals immune defense and subsequently facilitate invasion by any number of opportunistic pathogens. As algae are becoming increasingly abundant globally and as reefs are under the assault of an increasing number of stressors, it is urgent to determine whether algal-surface associated pathogens can migrate to coral surfaces, and whether this transmission is dependent upon coral health.

## Supporting Information

Table S1 Pairwise tests of 16S rRNA gene bacterial communities within separated coral and algal samples at Heron Island. (ND) Healthy coral, (AH) apparently healthy, (DL) disease lesion. * p<0.05; ns: not significant.
(DOCX)

Table S2 Pairwise tests of 16S rRNA gene bacterial communities within separated coral and algal samples at Los Roques. (ND) Healthy coral, (AH) apparently healthy, (T) transition and (DL) disease lesion. * p<0.05; ns: not significant.
(DOCX)

Table S3 Pairwise tests of bacterial diversity within separated coral and algal samples at Heron Island. (ND) Healthy coral, (AH) apparently healthy, (DL) disease lesion. * p<0.05; ns: not significant.
(DOCX)

Table S4 Pairwise tests of bacterial diversity within separated coral and algal samples at Los Roques. (ND) Healthy coral, (AH) apparently healthy, (T) transition and (DL) disease lesion. * p<0.05; ns: not significant.
(DOCX)

Video S1 **Time-lapse image of Yellow band Disease, showing the presence of ciliates at the disease lesion interface, although ciliates were not detected in the samples in this study, this illustrates that they are likely playing some kind of role YBD.**
(WMV)
