# Supplementary material for: Algae as Reservoirs for Coral Pathogens
Source: PLoS One. 2013 Jul 31;8(7):e69717. doi: 10.1371/journal.pone.0069717 (PMC3729954; doi:10.1371/journal.pone.0069717)
Supplement: Table S1 — Pairwise tests of 16S rRNA gene bacterial communities within separated coral and algal samples at Heron Island. (ND) Healthy coral, (AH) apparently healthy, (DL) disease lesion. * p<0.05; ns: not significant. (DOCX) [file pone.0069717.s001.docx]

**Table S1:**

|  | ND | AH | DL | *Padina australis* | *Hypnea sp.* | *Halimeda macroloba* | *Dictyota friabilis* | *Chlorodesmis fastigiata* | *Caulerpa cupressoides* | *Laurencia sp.* | *Hydroclathus clathrus* | *Caulerpa racemosa* | *Hincksia sp.* | *Sargassum polycystum* |
| --- | --- | --- | --- | --- | --- | --- | --- | --- | --- | --- | --- | --- | --- | --- |
| ND |  |  |  |  |  |  |  |  |  |  |  |  |  |  |
| AH | ns |  |  |  |  |  |  |  |  |  |  |  |  |  |
| DL | * | * |  |  |  |  |  |  |  |  |  |  |  |  |
| *Padina australis* | * | * | * |  |  |  |  |  |  |  |  |  |  |  |
| *Hypnea sp.* | * | * | * | ns |  |  |  |  |  |  |  |  |  |  |
| *Halimeda macroloba* | * | * | * | ns | ns |  |  |  |  |  |  |  |  |  |
| *Dictyota friabilis* | * | * | * | ns | ns | ns |  |  |  |  |  |  |  |  |
| *Chlorodesmis fastigiata* | * | * | * | ns | ns | ns | ns |  |  |  |  |  |  |  |
| *Caulerpa cupressoides* | * | * | * | ns | ns | ns | ns | ns |  |  |  |  |  |  |
| *Laurencia sp.* | * | * | * | ns | ns | ns | ns | ns | ns |  |  |  |  |  |
| *Hydroclathus clathrus* | * | * | * | * | * | * | * | * | * | * |  |  |  |  |
| *Caulerpa racemosa* | * | * | * | * | * | * | * | * | * | * | * |  |  |  |
| *Hincksia sp.* | * | * | * | * | * | * | * | * | * | * | * | * |  |  |
| *Sargassum polycystum* | * | * | * | * | * | * | * | * | * | * | * | * | * |  |
