# Supplementary material for: Algae as Reservoirs for Coral Pathogens
Source: PLoS One. 2013 Jul 31;8(7):e69717. doi: 10.1371/journal.pone.0069717 (PMC3729954; doi:10.1371/journal.pone.0069717)
Supplement: Table S4 — Pairwise tests of bacterial diversity within separated coral and algal samples at Los Roques. (ND) Healthy coral, (AH) apparently healthy, (T) transition and (DL) disease lesion. * p<0.05; ns: not significant. (DOCX) [file pone.0069717.s004.docx]

**Table S4:**

|  | ND | AH | T | DL | V Thin Turf | Thin Turf | *Dictyota sp.* | *Galaxaura sp.* | Thick Turf | V Thick Turf |
| --- | --- | --- | --- | --- | --- | --- | --- | --- | --- | --- |
| ND |  |  |  |  |  |  |  |  |  |  |
| AH | ns |  |  |  |  |  |  |  |  |  |
| T | * | ns |  |  |  |  |  |  |  |  |
| DL | * | ns | ns |  |  |  |  |  |  |  |
| V Thin Turf | ns | ns | ns | ns |  |  |  |  |  |  |
| Thin Turf | ns | ns | ns | ns | ns |  |  |  |  |  |
| *Dictyota sp.* | ns | ns | ns | ns | ns | ns |  |  |  |  |
| *Galaxaura sp.* | ns | ns | ns | ns | ns | ns | ns |  |  |  |
| Thick Turf | ns | * | * | * | ns | ns | ns | ns |  |  |
| V Thick Turf | ns | * | * | * | * | ns | ns | ns | ns |  |
